# Impact of Omega-3 on Endocannabinoid System Expression and Function, Enhancing Cognition and Behavior in Male Mice

**DOI:** 10.3390/nu16244344

**Published:** 2024-12-17

**Authors:** Maitane Serrano, Miquel Saumell-Esnaola, Garazi Ocerin, Gontzal García del Caño, Nagore Puente, Joan Sallés, Fernando Rodríguez de Fonseca, Marta Rodríguez-Arias, Inmaculada Gerrikagoitia, Pedro Grandes

**Affiliations:** 1 Department of Neurosciences, Faculty of Medicine and Nursing, University of the Basque Country UPV/EHU, 48940 Leioa, Spain; maitane.serrano@ehu.eus (M.S.); garazi.ocerin@ehu.eus (G.O.); nagore.puente@ehu.eus (N.P.); 2 Achucarro Basque Center for Neuroscience, Science Park of the UPV/EHU, 48940 Leioa, Spain; 3 Bioaraba, Neurofarmacología Celular y Molecular, 01006 Vitoria-Gasteiz, Spain; miquel.saumell@ehu.eus (M.S.-E.); gontzal.garcia@ehu.eus (G.G.d.C.); joan.salles@ehu.eus (J.S.); 4 Department of Pharmacology, Faculty of Pharmacy, University of the Basque Country UPV/EHU, 01006 Vitoria-Gasteiz, Spain; 5 Department of Neurosciences, Faculty of Pharmacy, University of the Basque Country UPV/EHU, 01006 Vitoria-Gasteiz, Spain; 6 Centro de Investigación Biomédica en Red de Salud Mental, 28029 Madrid, Spain; 7 Mental Health Clinical Management Unit, Institute of Biomedical Research of Málaga-IBIMA, Regional University Hospital of Málaga, 29010 Málaga, Spain; fernando.rodriguez@ibima.eu; 8 Department of Psychobiology, Faculty of Psychology, Universitat de València, 46010 Valencia, Spain; marta.rodriguez@uv.es

**Keywords:** CB1 receptor, hippocampus, memory, polyunsaturated fatty acids, synaptic plasticity

## Abstract

**Background/Objectives**: Omega-3 long-chain polyunsaturated fatty acids (PUFAs) support brain cell membrane integrity and help mitigate synaptic plasticity deficits. The endocannabinoid system (ECS) is integral to synaptic plasticity and regulates various brain functions. While PUFAs influence the ECS, the effects of omega-3 on the ECS, cognition, and behavior in a healthy brain remain unclear. **Methods and Results**: Here, we demonstrate that hippocampal synaptosomes from male mice fed an omega-3-rich diet exhibit increased levels of cannabinoid CB1 receptors (~30%), phospholipase C β1 (PLCβ1, ~30%), monoacylglycerol lipase (MAGL, ~30%), and cannabinoid receptor-interacting protein 1a (Crip1a, ~60%). Conversely, these synaptosomes show decreased levels of diacylglycerol lipase α (DAGLα, ~40%), synaptosomal-associated protein 25kDa (SNAP-25, ~30%), and postsynaptic density protein 95 (PSD-95, ~40%). Omega-3 intake also reduces Gαo and Gαi3 levels, though receptor-stimulated [^35^S]GTPγS binding remains unaffected. Stimulation of the medial perforant path (MPP) induced long-term potentiation (LTP) in omega-3-fed mice. This LTP was dependent on group I metabotropic glutamate receptors (mGluR), 2 arachidonoylglycerol (2-AG), CB1 receptors, *N*-type Ca^2+^ channels, and actin filaments. Behaviorally, omega-3-fed mice displayed reduced exploratory behavior and significantly improved object discrimination in the novel object recognition test (NORT). They also spent more time in open arms and exhibited reduced freezing time in the elevated plus maze (EPM), indicative of reduced anxiety-like behavior. **Conclusions**: Our findings suggest that omega-3 leverages the ECS to enhance brain function under normal conditions.

## 1. Introduction

The omega-3 fatty acids eicosapentaenoic acid (EPA) and docosahexaenoic acid (DHA), along with the omega-6 fatty acid arachidonic acid (AA), play essential roles in cell functions such as energy production, signal transduction, and membrane integrity [1,2,3,4,5]. Maintaining an adequate intake of omega-3, through diets rich in cold-water fatty fish, nuts, seeds, or plant oils, is crucial for overall health and homeostasis [1,2,3]. The brain is particularly enriched in DHA, with levels varying across different brain regions, neurons, and glial cell types, where it supports neurogenesis, neuronal migration, and synaptic pruning [2,4,6]. Moreover, DHA and EPA regulate gene expression and exert anti-inflammatory effects [2,7], in contrast to omega-6, which promotes inflammatory responses [3,5].

In recent decades, dietary patterns have shifted significantly, particularly in polyunsaturated fatty acid (PUFA) intake, leading to a disproportionate omega-6/omega-3 ratio of 20–30:1 in many Western countries [1,3]. In the brain, omega-3 intake enhances synaptic transmission in the hippocampus, supporting hippocampal-dependent memory functions [8,9]. Conversely, omega-3 deficiency, due to conditions such as excessive alcohol consumption [10,11,12], negatively affects synaptic plasticity and memory [4,6,13]. Notably, DHA supplementation restores omega-3 levels in the brain and rescues alcohol-impaired synaptic plasticity [12,13]. Additionally, low omega-3 levels impair endocannabinoid (eCB)-mediated synaptic plasticity in the brain [14].

The ECS is a complex signaling network that is extensively spread across the central nervous system (CNS) [15]. The specific localization of ECS components within different cell types and compartments plays a crucial role in their functional contribution to regulating brain functions. This system includes G protein-coupled cannabinoid receptors (CB1, CB2, and others), the primary eCBs, 2-AG and anandamide (AEA), along with the enzymes and transport mechanisms for eCB synthesis, degradation, and transport [15]. In excitatory synapses, neuronal membrane depolarization or activation of Gq-coupled G protein-coupled receptors (GPCRs) triggers the production of 2-AG. Synaptic glutamate spillover activates postsynaptic group I metabotropic glutamate receptors (mGluR5 and mGluR1), which are located at perisynaptic membrane sites. The most common pathway for 2-AG synthesis begins with the release of diacylglycerol (DAG) through the hydrolysis of membrane phosphoinositide 4,5-bisphosphate (PIP2) by phospholipase C β or δ. DAG is then hydrolyzed by diacylglycerol lipase (DAGL) α or β to produce 2-acylglycerols, including 2-AG. This endocannabinoid subsequently travels retrogradely to activate presynaptic CB1 receptors, inhibiting voltage-gated calcium channels and reducing neurotransmitter release. 2-AG plays a crucial role in various forms of synaptic plasticity through phasic endocannabinoid signaling [15]. Once its effects are exerted, 2-AG is degraded by monoacylglycerol lipase (MAGL), which catalyzes 2-AG hydrolysis into free fatty acids (such as arachidonic acid -AA-) and glycerol [15]. In the case of AEA, the primary biosynthetic pathway involves *N*-acyl phosphatidylethanolamine phospholipase D (NAPE-PLD), which hydrolyzes *N*-acyl phosphatidylethanolamine (NAPE) in cell membranes, serving as a precursor to AEA. AEA is subsequently degraded by the enzyme fatty acid amide hydrolase (FAAH). This enzyme catalyzes the hydrolysis of AEA within the cell, generating AA and ethanolamine. The CB1 receptor is one of the most abundant GPCRs in the brain. CB1 receptors display a broad but regionally variable expression pattern within the brain [15]. They interact with various proteins, particularly in the carboxy-terminal region, contributing to the complex regulation of cell-type-specific responses. Receptor binding involves interactions with Gα0 and Gαi3 proteins through a juxta-membrane domain in the *C*-terminal, as well as with Gαi1 and Gαi2 proteins via the third intracellular loop, which also interacts with Gs proteins. Also, cannabinoid receptor-interacting protein 1a (Crip1a), which is primarily expressed in presynaptic glutamatergic neurons, interacts with the distal portion of the CB1 carboxy-terminal. This interaction decreases receptor internalization and modulates intracellular signaling [16]. Crip1a mitigates the inhibitory effects of CB1 on *N*-type calcium channels by limiting CB1 receptor coupling to Gαo and Gαi3 proteins, thereby promoting neurotransmitter release [16,17,18]. Furthermore, Crip1a enhances CB1 receptor coupling in [^35^S]GTPγS assays using hippocampal homogenates [19] and favors CB1 coupling to Gαi1 and Gαi2 over Gαo and Gαi3 [19,20].

Despite the close relationship between omega-3 and the ECS [21], the effects of an omega-3-enriched diet on the hippocampal ECS and associated cognitive and behavioral functions under normal physiological conditions remain unexplored. In this study, we used a multidisciplinary approach, combining biochemical, anatomical, physiological, and behavioral techniques to examine these effects in the male mouse brain. Our findings reveal that omega-3 enhances CB1-dependent synaptic plasticity, correlating with changes in the ECS, cognitive performance, and behavior.

## 2. Materials and Methods

### 2.1. Ethical Statement

The protocols for animal care and use were approved by the Committee of Ethics for Animal Welfare of the University of the Basque Country (M20-2020-113; date of approval: 29 September 2020). They were also in agreement with the European Communities Council Directive of 22 September 2010 (2010/63/EU) and Spanish regulations (Real Decreto 53/2013, BOE 08-02-2013). The number of animals and amount of suffering were controlled and minimized.

### 2.2. Animal Treatment

**Figure 1:**
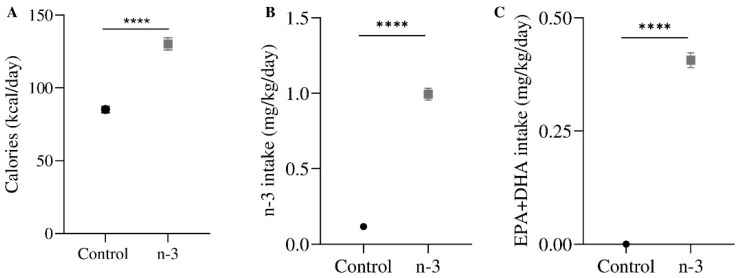
Dietary calorie consumption and omega-3 (*n*-3), EPA, and DHA intake. (**A**) Calorie intake (kcal/day) during young adulthood (PND 56–71). (**B**) Total *n*-3 intake (mg/kg/day). (**C**) EPA + DHA intake (mg/kg/day). Control group, n = 12; *n*-3 group, n = 12. Data are presented as mean ± SEM; Mann–Whitney test; **** *p* < 0.0001.

Eight-week-old C57BL/6J male mice (Janvier Labs, Le Genest-Saint-Isle, France) were randomly pair-housed and acclimatized for 4 days. Half of them were randomly fed on an omega-3-enriched diet (*n*-3 group) (SAFE, Augy, France) for two weeks during young adulthood (postnatal days (PNDs) 56 to 71) (Table 1). Twice a week, mice and food were weighed to measure calorie consumption (kcal/day: standard diet 85.14 ± 7.50 and omega-3 diet 130.20 ± 13.90, **** *p* < 0.0001), omega-3 (mg/kg/day: standard diet 0.12 ± 0.00 and omega-3 diet 0.99 ± 0.04, **** *p* < 0.0001) and particularly EPA and DHA intake (mg/kg/day: standard diet 0.00 ± 0.00 and omega-3 diet 0.41 ± 0.02, **** *p* < 0.0001) (Figure 1). Food was replaced once a week to avoid fat oxidation. Behavioral tests were performed on PNDs 68–70 coinciding with the last days of the dietary intake.

### 2.3. Synaptosomal Fractionation

Mice were sacrificed by cervical dislocation, brains were extracted, and hippocampi were dissected on ice and then stored at −80 °C until further use. Synaptosomes were prepared following a standard fractionation protocol [22] with minor modifications [23]. Considering that the yield of fractionation is approximately 1–1.5% (1–1.5 mg synaptosomal protein per 100 mg fresh tissue), pooled hippocampal tissue from at least six mice (about 160 mg fresh tissue weight) was used per fractionation procedure. Hippocampal tissue was thawed slowly on ice-cold 0.32 M sucrose at pH 7.4, containing 80 mM Na_2_HPO_4_ and 20 mM NaH_2_PO_4_ (sucrose phosphate buffer), and then it was homogenized in 10 volumes of sucrose/phosphate buffer, using a motor-driven Potter Teflon glass homogenizer (motor speed 800 rpm; 10 up- and downstrokes; cooled in an ice-water mixture throughout). A sample of the whole homogenate was stored at −80 °C for reference. The homogenate was centrifuged at 1000× *g* for 10 min, and the resulting supernatant (S1) was subsequently pelleted by centrifugation at 15,000× *g* for 30 min. The obtained pellet (P2, containing crude membranes) was re-suspended in 16 mL of 0.32 M sucrose/PB and transferred to a centrifugation tube containing 8 mL of sucrose/PB solution (1.2 M) placed at the bottom of the centrifuge tube, and the homogenate was centrifuged at 180,000× *g* for 30 min. The material collected at the gradient interface was removed, diluted to 16 mL with 0.32 M sucrose/PB, layered onto 8 mL of 0.8 M sucrose/PB, and centrifuged again at 180,000× *g* for 30 min. The resulting synaptosomal pellet was re-suspended in PB, and aliquots were centrifuged at 40,000× *g* for 30 min. After aspiration of the supernatant, the synaptosomal pellets were frozen at −80 °C for storage. Protein content was measured using Bio-Rad dye reagent with a bovine globulin standard.

### 2.4. Western Blotting

Western blotting was performed as previously described [24], with minor adjustments. Whole homogenates and synaptosomal fractions were heated for 5 min at 60 °C in a urea-denaturing buffer (20 mM Tris-HCl, pH 8.0, 12% glycerol, 12% urea, 5% DTT, 2% SDS, and 0.01% bromophenol blue). Denatured proteins were separated by SDS–polyacrylamide gel electrophoresis (SDS–PAGE) on 10% gradient gels using the Mini Protean II system (Bio-Rad, Hercules, CA, USA). Proteins were then transferred onto polyvinylidene fluoride (PVDF) membranes (Amersham Bioscience, Buckinghamshire, UK) using the Mini TransBlot transfer unit (Bio-Rad, Hercules, CA, USA) at 30 V overnight at 4 °C. Membranes were blocked for 1 h at room temperature (RT) with 5% non-fat dry milk in phosphate-buffered saline (PBS) containing 0.5% bovine serum albumin (BSA) and 0.2% Tween-20. Primary antibodies, diluted in blocking buffer, were then applied (Table 2), followed by incubation with horseradish peroxidase-conjugated secondary antibodies (diluted 1:10,000 in blocking buffer) for 2 h at RT. Immunoreactive bands were visualized using the enhanced chemiluminescence detection system, following the manufacturer’s instructions (Amersham Bioscience, Buckinghamshire, UK), and images were captured on an ImageQuant 350 imager. A color pre-stained broad-range protein ladder (MB090, NZYtech, Lisbon, Portugal) was used to estimate the molecular mass of individual bands. Densitometric analysis of digital images was performed using ImageJ software (NIH, Bethesda, MD, USA), with data from omega-3 mice normalized to the linear regression slope of control samples and expressed as a ratio.

### 2.5. [^35^S]GTPγS Binding Assays

We followed a published protocol [25] with minor modifications. Briefly, 5 µg of synaptosomal fractions were incubated at 30 °C for 2 h in [^35^S]GTPγS incubation buffer (0.5 nM [^35^S]GTPγS, 1 mM EGTA, 3 mM MgCl_2_, 100 mM NaCl, 0.2 mM DTT, 50 μM GDP, 0.5% BSA [fatty acid-free], and 50 mM Tris-HCl, pH 7.4). Basal binding was defined as specific [^35^S]GTPγS binding in the absence of an agonist. To measure receptor-stimulated [^35^S]GTPγS binding, the CB1 receptor agonist CP55,940 (1 nM–10 µM) was added, while nonspecific binding was assessed with 10 µM unlabeled GTPγS. Reactions were terminated by rapid vacuum filtration through Whatman GF/B glass fiber filters, and bound radioactivity was quantified using liquid scintillation spectrophotometry. The concentration-dependent increase in specific [^35^S]GTPγS binding induced by CP55,940 was expressed as a percentage of the basal, unstimulated binding level and analyzed via non-linear regression using the four-parameter Hill equation to determine the maximum percentage increase (Emax) and the concentration eliciting half-maximal binding (pEC50).

### 2.6. Slice Preparation and Extracellular Field Recordings

Mice (PND 67–71) were anesthetized with isoflurane, after which their brains were quickly removed and placed in a chilled sucrose-based solution (87 mM NaCl, 75 mM sucrose, 25 mM glucose, 7 mM MgCl_2_, 2.5 mM KCl, 0.5 mM CaCl_2_, and 1.25 mM NaH_2_PO_4_) at 4 °C. Coronal sections (300 μm thick) were prepared using a vibratome (Leica VT 1000s, Leica Biosystems, Barcelona, Spain) and maintained at 35 °C and then superfused in the recording chamber (2 mL/min) with artificial cerebrospinal fluid (130 mM NaCl, 11 mM glucose, 1.2 mM MgCl_2_, 2.5 mM KCl, 2.4 mM CaCl_2_, 1.2 mM NaH_2_PO_4_, and 23 mM NaHCO_3_, equilibrated with 95% O_2_/5% CO_2_). Picrotoxin (100 μM) was added to the superfusion medium to inhibit GABAa receptors, and all other drugs were added to their final concentrations (Table 3). Extracellular field recordings were obtained by positioning the stimulation electrode in the middle third and the recording pipette in the inner third of the dentate molecular layer [26]. After recording a stable baseline, low-frequency stimulation (LFS; 10 min at 10 Hz) was applied to induce CB1 receptor-dependent long-term excitatory synaptic plasticity, with and without drugs present. The area of field excitatory postsynaptic potentials (fEPSPs) was measured [26]. The extent of long-term synaptic plasticity following LFS was calculated as the percentage change between baseline (averaged excitatory responses during the 10 min preceding LFS) and the last 10 min of stable responses in the 30-min period after LFS [26]. Slices used for recordings (n) were obtained from at least three omega-3 and three control mice.

### 2.7. Behavioral Tests

Behavioral tests were conducted from PND 68 to PND 70 with different cohorts of animals of at least twelve animals per experimental group. Mice were moved into the behavioral testing room 45 min prior to testing, in a room with lights on and a temperature of 22 °C.

#### 2.7.1. Novel Object Recognition Test

Long-term recognition memory was evaluated in the L-maze. On the first day (PND 68), mice were habituated to the maze for 9 min. On the acquisition day (PND 69), two identical objects were placed at opposite ends of the maze. Mice were introduced at the maze’s corner and allowed to explore the objects for 9 min. On the final day (PND 70), one of the familiar objects was replaced with a novel object, and the mice were given 9 min to explore. Total exploration time on both acquisition and test days was recorded, and the discrimination index (DI) was calculated as follows: DI = (time spent exploring the novel object − time spent exploring the familiar object)/total exploratory time). A DI of ≥0.3 indicates good recognition memory [27].

#### 2.7.2. Anxiety Tests

Anxiety-related behaviors were assessed over three consecutive days using the open field (OF), elevated plus maze (EPM), and light/dark box (LDB) tests [28].

##### Open Field (OF) Test

Mice were individually placed in a square open area (40 cm in length × 45 cm in height × 40 cm in width), divided into a 28-cm inner center zone and a 6-cm outer safe zone. Each mouse was put into the center and allowed to explore freely for 5 min. The time spent in the center zone and the latency to move into the safe zone were recorded.

##### Elevated Plus Maze (EPM) Test

Each mouse was individually placed at the center of an X-shaped maze, containing two open and two closed arms, and allowed to explore for 5 min. Time spent in the open arms and freezing time were measured.

##### Light/Dark Box (LDB) Test

Mice were individually placed in a light box (390 lux) connected to a dark box and were allowed to explore both boxes freely for 5 min. The time spent in the light zone and the number of entries into the light zone were recorded.

### 2.8. Statistical Analysis

Statistical analyses were performed using GraphPad Prism 8 (GraphPad Software, LLC, Boston, MA, USA; RRID: SCR_002798). Data normality was assessed using the Shapiro–Wilk test. Depending on the results, either parametric (*t*-test or ratio *t*-test) or nonparametric (Mann–Whitney test) analyses were conducted as appropriate. All values are reported as mean ± SEM.

## 3. Results

### 3.1. Omega-3 Enrichment Alters the Endocannabinoid System (ECS)

**Figure 2:**
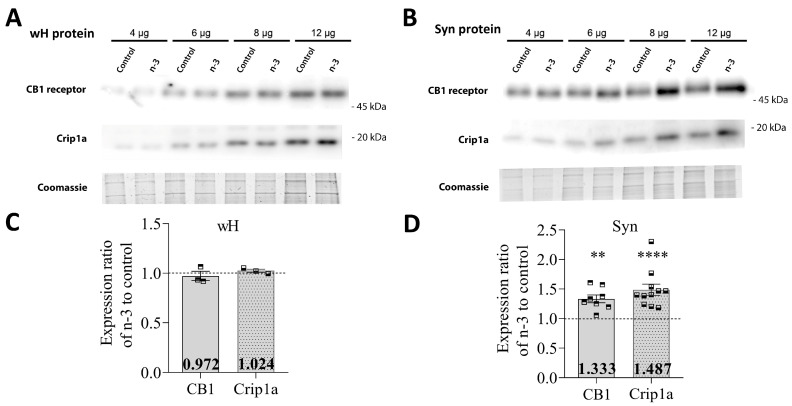
CB1 and Crip1a protein expression in hippocampal whole homogenates and synaptosomes. (**A**,**B**) Representative Western blots of CB1 and Crip1a expression carried out by immunoblotting increasing amounts of total protein from hippocampal whole homogenates (wH) or synaptosomes (Syn). The total protein loading was checked by Coomassie Brilliant Blue gel staining. Protein migration was consistent with their expected molecular mass (CB1, 52.8 kDa; Crip1a, 18.6 kDa). The molecular weights depicted correspond to the signal of the standard markers. (**C**) CB1 and Crip1a expression in whole homogenates. (**D**) CB1 and Crip1a expression in synaptosomes. Data are presented as mean ± SEM (see Table 4), with squares representing individual experimental values, using a synaptosomal or whole homogenate fraction obtained from two fractionation procedures and including hippocampal pools from at least six adult mice per fractionation procedure. Statistical analysis: paired ratio *t*-test; ** *p* < 0.01, **** *p* < 0.0001.

In whole hippocampal homogenates, omega-3 enrichment did not significantly alter expression levels of the CB1 receptor (omega-3: 0.972 ± 0.046; t = 0.65, *p* = 0.5893 vs. control) or Crip1a (omega-3: 1.024 ± 0.016; t = 1.44, *p* = 0.2855 vs. control) between groups (Figure 2A,C). However, in the hippocampal synaptosomes of omega-3 supplemented mice, CB1 expression showed a significant increase (omega-3: 1.333 ± 0.065; t = 5.40, *p* < 0.0010 vs. control), as did Crip1a expression (omega-3: 1.487 ± 0.095; t = 6.63, *p* < 0.0001 vs. control) (Figure 2B,D).

**Figure 3:**
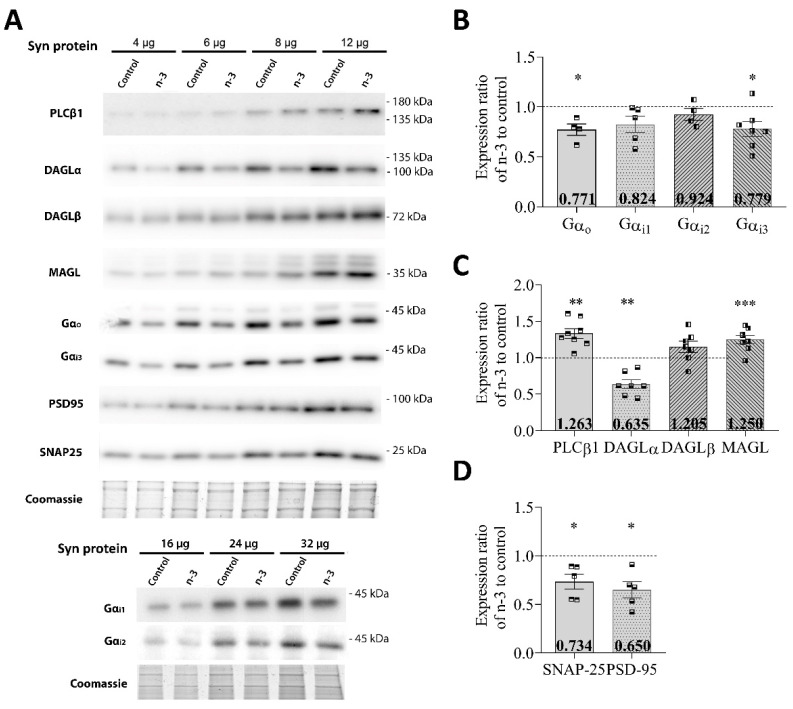
Relative expression of Gαi/o subunits, main 2-AG-related enzymes, and synaptic proteins in hippocampal synaptosomes (Syn). (**A**) Representative Western blots carried out by immunoblotting increasing amounts of hippocampal synaptosomes. The total protein loading was checked by Coomassie Brilliant Blue gel staining. Protein migration was consistent with their expected molecular mass (PLCβ1, 138.3 kDa; DAGLα, 115.3 kDa; DAGLβ, 73.9 kDa; MAGL, 33.3 kDa; Gαo 40.1 kDa; Gαi1, 40.5 kDa; Gαi2, 40.4 kDa; Gαi3, 40.5 kDa; PSD-95, 80.4 kDa; SNAP-25, 23.3 kDa). The molecular weights depicted correspond to the signal of the standard markers. (**B**) Gαo, Gαi1, Gαi2, and Gαi3 expression in synaptosomes. (**C**) PLCβ1, DAGLα, DAGLβ, and MAGL expression in synaptosomes. (**D**) SNAP-25 and PSD-95 expression in synaptosomes. Data are presented as mean ± SEM (see Table 4) with squares representing individual experimental values, using synaptosome membranes obtained from two fractionation procedures and including hippocampal pools from at least six adult mice per fractionation procedure. Statistical analysis: Paired ratio *t*-test; * *p* < 0.05, ** *p* < 0.01, *** *p* < 0.001.

Omega-3 enrichment also notably affected the expression of Gα protein subunits in synaptosomal fractions obtained from the hippocampus. Specifically, there was a significant reduction in Gαo (omega-3: 0.7719 ± 0.057; t = 3.47, *p* = 0.0405 vs. control) and Gαi3 (omega-3: 0.779 ± 0.075; t = 2.87, *p* = 0.0285 vs. control) (Figure 3A,B and Table 4), while no significant changes were observed in Gαi1 (omega-3: 0.824 ± 0.081; t = 2.02, *p* = 0.1129 vs. control) or Gαi2 (omega-3: 0.924 ± 0.059; t = 1.31, *p* = 0.2810 vs. control) (Figure 3A,B and Table 4).

Regarding the 2-AG synthesizing enzymes, omega-3 intake resulted in a significant increase in PLCβ1 expression (omega-3: 1.263 ± 0.054; t = 4.13, *p* = 0.0026 vs. control) and a significant decrease in DAGLα expression (omega-3: 0.635 ± 0.059; t = 5.14, *p* = 0.0021 vs. control), while DAGLβ levels remained unchanged (omega-3: 1.205 ± 0.064; t = 1.73, *p* = 0.1350 vs. control). MAGL expression was also significantly elevated in omega-3-enriched mice compared to controls (omega-3: 1.250 ± 0.055; t = 7.71, *p* = 0.0002 vs. control) (Figure 3A,C and Table 4).

Lastly, synaptic protein levels were affected, with SNAP-25 (omega-3: 0.734 ± 0.076; t = 3.02, *p* = 0.0390 vs. control) and PSD-95 (omega-3: 0.650 ± 0.082; t = 3.57, *p* = 0.0234 vs. control) both significantly decreasing in omega-3 mice compared to controls (Figure 3A,D and Table 4).

### 3.2. Effect of Omega-3 Supplementation on CB1 Receptor Coupling to Gαi/o

**Figure 4:**
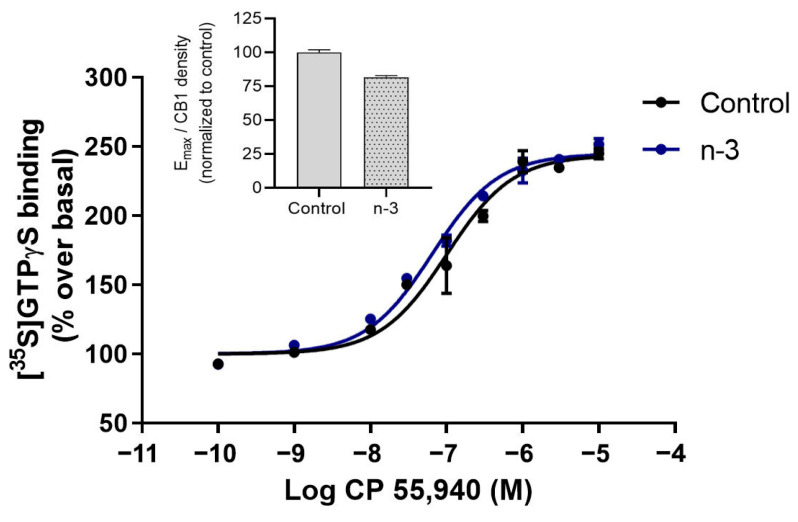
CB1 receptor coupling to Gαi/o proteins in hippocampal synaptosomes from control and omega-3 (*n*-3) mice. Concentration–response curves for CP 55,940-stimulated [^35^S]GTPγS binding. Curves represent mean ± SEM from triplicate data points of three independent experiments. Emax values are expressed as % specific [^35^S]GTPγS bound of basal. The inset shows the ratio of Emax to CB1 receptor expression values (determined by Western blot).

CB1 receptor coupling to Gαi/o proteins did not show a significant difference in Emax (omega-3: 159.00 ± 8.56; control: 156.90 ± 12.80; t = 0.34, *p* = 0.7689; Figure 4) or pEC50 values (omega-3: 7.13 ± 0.08; control: 7.03 ± 0.07; t = 2.22, *p* = 0.0782) between synaptosomal membranes from control and omega-3 supplemented mice. In addition, the efficiency of CB1 receptor coupling to Gαi/o was reduced in omega-3 mice, as indicated by increased CB1 expression without corresponding changes in Emax and pEC50 values (Figure 4, inset) (Table 5).

### 3.3. Omega-3 Enrichment Enhances Synaptic Potentiation

**Figure 5:**
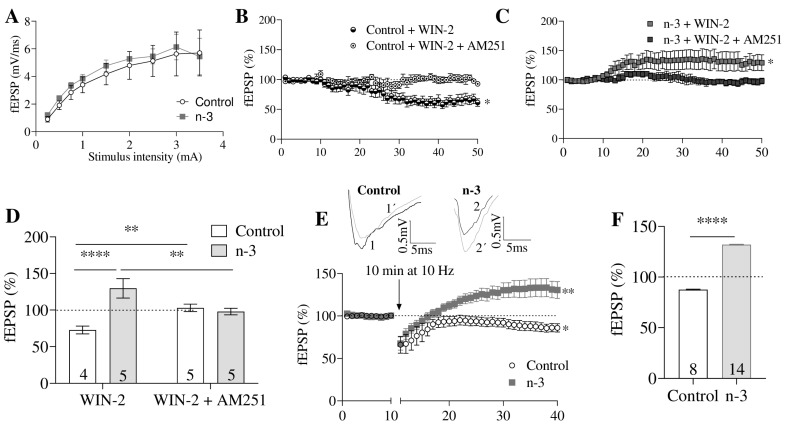
Omega-3 (*n*-3)-enriched diet induces CB1-mediated excitatory synaptic transmission and MPP-LTP. (**A**) Input–output curves: mean fEPSP areas (mV/ms) plotted against stimulation intensities in hippocampal slices from control (gray squares, n = 8) and *n*-3 (black circles, n = 14) mice. Data are mean ± SEM, analyzed by unpaired *t*-test. (**B**) Time course plot in control mice: WIN-2 (5 μM) (black and white circles) decreases fEPSP, while WIN-2 (5 μM) + AM251 (4 μM) (white circles) has no effect. Data are mean ± SEM; unpaired *t*-test, * *p* < 0.05 vs. baseline. (**C**) In *n*-3 mice, WIN-2 (5 μM) (gray squares) increases fEPSP, whereas WIN-2 (5 μM) + AM251 (4 μM) (black squares) shows no effect. Data are mean ± SEM; unpaired *t*-test, * *p* < 0.05 vs. baseline. (**D**) Summary bar graph for control + WIN-2 (5 μM), *n*-3 + WIN-2 (5 μM), control + WIN-2 (5 μM) + AM251 (4 μM), and *n*-3 + WIN-2 (5 μM) + AM251 (4 μM). Numbers within the bars are individual experiments; data are mean ± SEM, analyzed by one-way ANOVA with Dunn’s multiple comparisons test, ** *p* < 0.01, **** *p* < 0.0001. (**E**) **Top**: averaged fEPSP traces showing the effect of LFS (10 min, 10 Hz) over the last 10 min. LFS induces MPP-LTD in control mice (gray line) and MPP-LTP in *n*-3 mice (gray line). **Bottom**: LFS triggers MPP-LTD in control (white circles) and MPP-LTP in *n*-3 (gray squares). Data are mean ± SEM; Student’s *t*-test, * *p* < 0.05, ** *p* < 0.01 vs. baseline. (**F**) Summary bar graph of MPP-LTD and MPP-LTP. Numbers within the bars are individual experiments; data are mean ± SEM; Student’s *t*-test, **** *p* < 0.0001.

The input–output relationship between the fEPSP slope and stimulus intensity was similar in control and omega-3-enriched mice (control: n = 8; omega-3: n = 14; t = 0.49, *p* = 0.6313; Figure 5A). In omega-3 mice, the application of WIN 55,212-2 (WIN-2; 5 µM) significantly increased excitatory synaptic transmission at MPP synapses (n = 5, 124.30 ± 0.46% of fEPSP; t = 2.45, *p* = 0.0400 vs. baseline), an effect that was reversed by AM251 (4 µM; n = 5, 101.10 ± 0.37% of fEPSP; t = 0.48, *p* < 0.05 vs. baseline) (Figure 5C,D). In contrast, in control mice, WIN-2 (5 µM) reduced excitatory synaptic transmission at MPP synapses (n = 4, 72.80 ± 0.78% of fEPSP; t = 4.12, *p* = 0.0034 vs. baseline), which was similarly reversed by AM251 (4 µM; n = 5, 99.74 ± 1.21% of fEPSP; t = 0.64, *p* < 0.05 vs. baseline) (Figure 5B,D).

**Figure 6:**
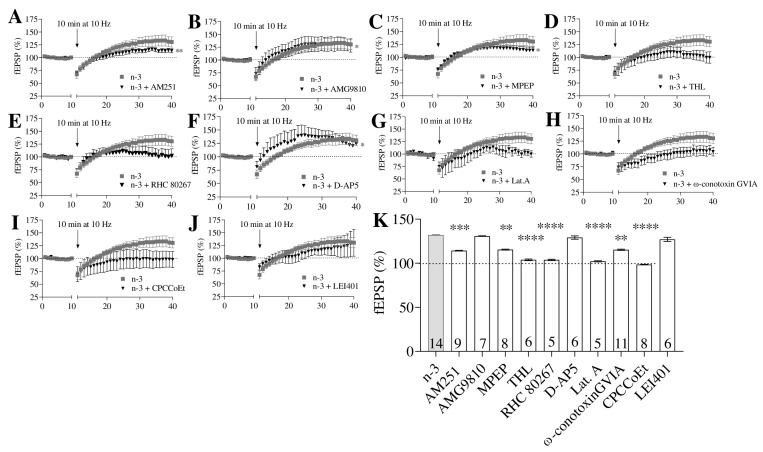
MPP-LTP in omega-3 (*n*-3) mice is mediated by CB1, group I mGluRs, and 2-AG. Hippocampal slices were obtained from n = 14 mice. (**A**–**J**) Effects of pharmacological inhibitors on MPP-LTP in *n*-3 mice, shown with black triangles: (**A**) AM251 (4 µM): paired *t*-test, ** *p* < 0.01 vs. baseline. (**B**) AMG9810 (3 µM): Wilcoxon test, * *p* < 0.05 vs. baseline. (**C**) MPEP (10 µM): paired *t*-test, * *p* < 0.05 vs. baseline. (**D**) THL (10 µM): paired *t*-test, *p* > 0.05 vs. baseline. (**E**) RHC 80,287 (100 µM): paired *t*-test, *p* > 0.05 vs. baseline. (**F**) D-AP5 (50 µM): Wilcoxon test, * *p* < 0.05 vs. baseline. (**G**) LAT-A (500 µM): paired *t*-test, *p* > 0.05 vs. baseline. (**H**) ω-Conotoxin GVIA (1 µM): paired *t*-test, *p* > 0.05 vs. baseline. (**I**) CPCCoEt (50 µM): paired *t*-test, *p* > 0.05 vs. baseline. (**J**) LEI401 (10 µM): Wilcoxon test, *p* > 0.05 vs. baseline. (**K**) Summary bar graph displaying results for: *n*-3, *n*-3 + AM251 (4 µM), *n*-3 + AMG9810 (3 µM), *n*-3 + MPEP (10 µM), *n*-3 + THL (10 µM), *n*-3 + RHC 80,287 (100 µM), *n*-3 + D-AP5 (50 µM), *n*-3 + LAT-A (500 µM), *n*-3 + ω-Conotoxin GVIA (1 µM), *n*-3 + CPCCoEt (50 µM), and *n*-3 + LEI401 (10 µM). Numbers within the bars indicate individual experiments; data are expressed as mean ± SEM. Statistical analysis: Dunn’s test; ** *p* < 0.01, *** *p* < 0.001, **** *p* < 0.0001.

Low-frequency stimulation (LFS) induced long-term potentiation (LTP) at MPP synapses in omega-3-enriched mice (n = 14), 132.10 ± 0.36% of fEPSP; t = 3.20, *p* = 0.0036 vs. baseline), contrasting with the long-term depression (LTD) observed in control mice (n = 8, 87.62 ± 5.19% of fEPSP; t = 2.39, *p* = 0.0315 vs. baseline) (Figure 5E,F). This MPP-LTP was partially blocked by AM251 (4 µM; n = 9, 114.3 ± 0.41% of fEPSP; *p* = 0.0037 vs. baseline; *p* = 0.0016 vs. omega-3; Figure 6A,K) and MPEP (10 µM; n = 8, 115.5 ± 0.56% of fEPSP; *p* = 0.0416 vs. baseline; *p* = 0.0068 vs. omega-3; Figure 6C,K), suggesting the involvement of CB1 and mGluR5.

Additionally, MPP-LTP was completely inhibited by THL (10 µM; n = 6, 103.9 ± 0.92% of fEPSP; *p* = 0.6835 vs. baseline; *p* < 0.0001 vs. omega-3; Figure 6D,K), RHC 80,267 (100 µM; n = 5, 103.8 ± 0.78% of fEPSP; *p* = 0.7936 vs. baseline; *p* < 0.0001 vs. omega-3; Figure 6E,K), latrunculin A (LAT-A, 500 µM; n = 5, 102.2 ± 0.64% of fEPSP; *p* = 0.7005 vs. baseline; *p* < 0.0001 vs. omega-3; Figure 6G,K), ω-conotoxin GVIA (1 µM; n = 11, 115.3 ± 0.74% of fEPSP; *p* = 0.1904 vs. baseline; *p* = 0.0055 vs. omega-3; Figure 6H,K), and CPCCoEt (50 µM; n = 8, 98.60 ± 0.27% of fEPSP; *p* = 0.9407 vs. baseline; *p* < 0.0001 vs. omega-3; Figure 6I,K), indicating the involvement of 2-AG, presynaptic actin assembly, *N*-type Ca^2+^ channels, and mGluR1.

In contrast, MPP-LTP was unaffected by AMG9810 (3 µM; n = 7, 130.8 ± 0.38% of fEPSP; *p* = 0.0156 vs. baseline; *p* = 0.6792 vs. omega-3; Figure 6B,K), D-AP5 (50 µM; n = 6, 129.2 ± 1.94% of fEPSP; *p* = 0.0313 vs. baseline; *p* = 0.5991 vs. omega-3; Figure 6F,K), and LEI401 (10 µM; n = 6, 127.1 ± 2.33% of fEPSP; *p* = 0.1563 vs. baseline; *p* = 0.4042 vs. omega-3; Figure 6J,K). These findings suggest that transient receptor potential vanilloid 1 (TRPV1), ionotropic *N*-methyl-D-aspartate (NMDA) receptors, and AEA do not contribute to this form of synaptic plasticity in omega-3 supplemented mice.

### 3.4. Omega-3 Enrichment Enhances Long-Term Recognition Memory

**Figure 7:**
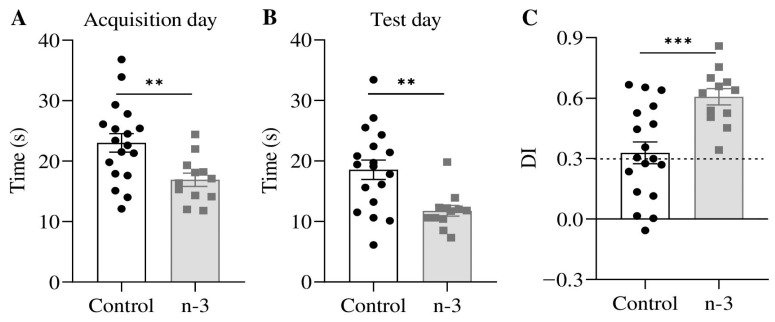
Novel object recognition test. (**A**) Total exploration time (s) spent with objects on the acquisition day (unpaired *t*-test). (**B**) Total exploration time (s) spent with objects on the test day (Mann–Whitney test). (**C**) Discrimination index on the test day (unpaired *t*-test). Control group, n = 18; *n*-3 group, n = 12. Black circles and gray squares represent individual values. Data are presented as mean ± SEM; ** *p* < 0.01, *** *p* < 0.001.

Mice on the omega-3-enriched diet spent significantly less time exploring objects on both the acquisition day (omega-3: 16.92 ± 1.10 s; control: 23.02 ± 1.54 s; t = 2.91, *p* = 0.0070; Figure 7A) and the test day (omega-3: 11.76 ± 0.89 s; control: 18.56 ± 1.61 s; U = 45, *p* = 0.0067; Figure 7B). Furthermore, the DI was significantly improved in omega-3-enriched mice (omega-3: 0.61 ± 0.04; control: 0.33 ± 0.05; t = 3.74, *p* = 0.0008; Figure 7C), indicating enhanced recognition memory with omega-3 supplementation.

### 3.5. Omega-3 Supplementation Induces Anxiolytic-like Effects

**Figure 8:**
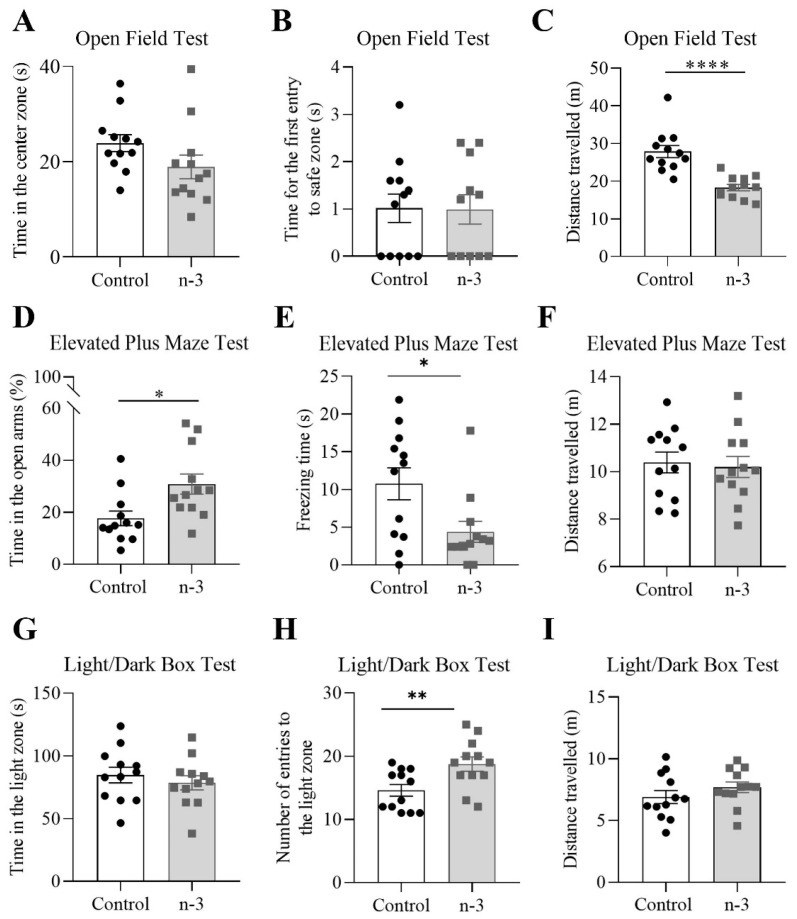
Anxiety- like behavior analysis using the OF, EPM, and LDB tests. (**A**) Total time (s) spent in the center zone of the open field (OF) maze (unpaired *t*-test). (**B**) Time (s) spent in the center zone before first entry into the safe zone of the OF (Mann–Whitney test). (**C**) Distance traveled (m) in the OF (unpaired *t*-test). (**D**) Percentage of time (%) spent in the open arms of the elevated plus maze (EPM) (unpaired *t*-test). (**E**) Freezing time (s) during the EPM test (Mann–Whitney test). (**F**) Distance traveled (m) in the EPM (unpaired *t*-test). (**G**) Total time (s) spent in the light zone of the light/dark box (LDB) (unpaired *t*-test). (**H**) Number of entries into the light zone of the LDB (Mann–Whitney test). (**I**) Distance traveled (m) in the LDB (unpaired *t*-test). Control group, n = 12; *n*-3 group, n = 12. Black circles and gray squares represent individual values. Data are presented as mean ± SEM; * *p* < 0.05, ** *p* < 0.01, **** *p* < 0.0001.

In the OF test, there were no significant differences between the control and omega-3 groups in time spent in the center (omega-3: 18.90 ± 2.48 s; control: 23.91 ± 1.76 s; t = 1.65, *p* = 0.1140; Figure 8A) or in latency to first safe zone entry (omega-3: 0.99 ± 0.31 s; control: 1.02 ± 0.30 s; U = 65.50, *p* = 0.9841; Figure 8B). However, in the EPM test, omega-3 supplemented mice spent more time in the open arms (omega-3: 30.85 ± 13.45%; control: 17.66 ± 9.82%; t = 2.74, *p* = 0.0119; Figure 8D) and showed reduced freezing time (omega-3: 4.40 ± 1.40 s; control: 10.75 ± 2.12 s; U = 35.00, *p* = 0.0330; Figure 8E).

In the LDB test, while there was no difference in time spent in the light zone (omega-3: 78.63 ± 5.63 s; control: 84.72 ± 6.21 s; t = 0.73, *p* = 0.4757; Figure 8G), omega-3 supplemented mice made significantly more entries into the light zone (omega-3: 18.75 ± 1.13 entries; control: 14.58 ± 0.92 entries; U = 27.50, *p* = 0.0082; Figure 8H).

For the distance traveled, the omega-3 group showed reduced movement in the OF test (omega-3: 18.29 ± 2.92 m; control: 27.86 ± 5.59 m; t = 5.26, *p* < 0.0001; Figure 8C), with no significant differences observed in the EPM (omega-3: 10.20 ± 1.53 m; control: 10.39 ± 1.52 m; t = 0.30, *p* = 0.7653; Figure 8F) or LDB tests (omega-3: 7.69 ± 1.50 m; control: 6.90 ± 1.83 m; t = 1.15, *p* = 0.2622; Figure 8I).

## 4. Discussion

This study demonstrates that omega-3 intake during young adulthood (1) changes the ECS in the hippocampus, (2) influences excitatory synaptic transmission and plasticity at MPP synapses, which convey spatial memory information and context-dependent learning to the dentate gyrus, and (3) enhances long-term recognition memory and reduces anxiety-like behaviors.

We observed an approximately 40% decrease in DAGLα, the primary enzyme for 2-AG synthesis, in the hippocampal synaptosomes of omega-3-enriched mice. This finding aligns with previous reports of reduced brain 2-AG levels in animals fed omega-3-enriched diets [29,30]. These modifications were accompanied by a ~30% increase in MAGL, the primary enzyme for 2-AG degradation. This suggests a regulatory effect on both 2-AG synthesis and degradation, potentially impacting CB1 receptor expression and function under omega-3-enriched conditions.

Accordingly, we found a notable increase in CB1 (~30%) and Crip1a expression (~60%) in the hippocampal synaptosomes of supplemented mice compared to controls. Interestingly, these changes were not detected in whole hippocampal homogenates, suggesting that CB1 and Crip1a expression changes are limited to synaptic compartments after omega-3 intake. Chronic exposure to cannabinoids typically leads to CB1 receptor desensitization through G protein uncoupling or internalization, with exogenous cannabinoids prompting receptor accumulation at extrasynaptic sites [31,32,33]. Crip1a, a presynaptic protein that binds the CB1 receptor carboxy terminus, reduces receptor internalization and intracellular signaling, which ultimately promotes neurotransmitter release [16,17,18]. In previous studies on hippocampi from TRPV1 knockout mice, we observed increased Crip1a and Gαi/o subunit levels in synaptosomes, as well as potentiated [^35^S]GTPγS binding following CB1 receptor agonism, despite decreased synaptosomal CB1 receptor expression [34]. Furthermore, we observed a significant increase in the proportion (~30%) and number (~50%) of CB1-positive excitatory synaptic terminals and their immunolabeling intensity [35].

Given the significant increases in CB1 receptor and Crip1a expression in hippocampal synaptosomes from omega-3 supplemented mice, we conducted [^35^S]GTPγS binding assays to determine the impact on CB1 coupling to Gαi/o proteins. The reduction in Gαo and Gαi3 expression induced by the omega-3-enriched diet, combined with the substantial increase in Crip1a, may counterbalance the expected increase in CB1 receptor signaling. In fact, the decrease in Gαo and Gαi3 subunits in the omega-3 group was not associated with changes in the maximal efficacy (Emax) or potency (pEC50) of stimulated [^35^S]GTPγS binding, suggesting that omega-3 supplementation limits CB1 receptor signaling. Crip1a has been shown to preferentially enhance CB1 receptor coupling to Gαi1 and Gαi2 over Gαo and Gαi3 [36], implying that the relative availability of each Gαi/o subunit could differentially modulate CB1 receptor activation, possibly in opposing directions [18].

Research indicates that Gαo is the most abundant subunit in glutamatergic and GABAergic synaptic terminals in the mouse hippocampus, followed by Gαi1 > Gαi2 > Gαi3 [37]. Therefore, the increase in Crip1a in omega-3 mice might act as a net negative regulator of CB1 receptor coupling to Gαi/o proteins. This interpretation should be approached with caution, as studies in cellular models report a negative effect of Crip1a on CB1 receptor coupling to Gαi/o [20], whereas Crip1a has been shown to enhance receptor coupling in [^35^S]GTPγS assays in whole hippocampal homogenates [19].

These modifications in CB1 receptor intracellular signaling appear to influence synaptic plasticity. In our study with mice fed an omega-3-enriched diet, we observed that LFS of the dentate MPP synapses, instead of inducing LTD as previously reported [26,38,39], triggered an eCB-dependent increase in the fEPSP slope that was sustained for the duration of the experiment (30 min post-LFS). Interestingly, this MPP-LTP in omega-3-fed mice required the same components typically involved in MPP-LTD: group I mGluRs, 2-AG, and CB1 receptors [26]. Additionally, *N*-type Ca^2+^ channels and presynaptic actin remodeling were necessary [40,41,42].

The signaling mechanisms by which 2-AG and CB1 receptors facilitate LTP following omega-3 supplementation remain largely unexplored. One plausible explanation for the shift toward synaptic potentiation may involve changes in 2-AG availability under omega-3 dietary conditions. Prolonged CB1 activation by low 2-AG levels promotes protein kinase A (PKA) inhibition, whereas activation by high 2-AG over a short period stimulates PKA activity [43]. Thus, both the concentration and timing of 2-AG are critical in modulating the shift from LTD to LTP [44]. Furthermore, limited availability of Gαi/o proteins has been associated with a shift in CB1 receptor coupling to Gs proteins, leading to increased cyclic AMP (cAMP) [45,46]. Notably, DHA has been shown to alter Gs levels, thereby impacting cAMP [47].

Endocannabinoid-mediated long-term synaptic plasticity may occur via the cAMP/PKA pathway [48,49], similar to LTP at hippocampal mossy fiber synapses [50]. Indeed, a DHA metabolite has been found to increase cAMP levels [51,52], thereby activating the cAMP/PKA pathway and subsequently enhancing hippocampal LTP [53,54].

MPP-LTP in mice on an omega-3-enriched diet requires *N*-type Ca^2+^ channels, which are known to enhance neurotransmitter release efficiency [55]. Notably, Crip1a attenuates the inhibitory effect of CB1 on *N*-type Ca^2+^ influx [17]. We also observed an approximately 30% decrease in synaptosomal SNAP-25 levels in omega-3-fed mice. SNAP-25 inhibits presynaptic P-, Q-, and L-type voltage-gated calcium channels [56] and reduces Ca^2+^ effects at excitatory synapses [57]. Therefore, the substantial increase in Crip1a and the observed reduction in SNAP-25 due to the omega-3-enriched diet would likely promote glutamate release at MPP synapses.

The enhancement in neurotransmitter release that mediates LTP at lateral perforant path synapses has been shown to require actin cytoskeleton remodeling [40,41]. Thus, the inhibition of MPP-LTP by LAT-A observed in omega-3-fed mice also suggests a role for actin polymerization in MPP synaptic potentiation. Collectively, the CB1-associated presynaptic molecular events triggered by the omega-3 diet upon LFS result in a switch from CB1-dependent LTD to LTP at excitatory MPP synapses [34]. However, CB1 receptor antagonism did not completely block synaptic potentiation, suggesting additional mechanisms are involved.

One possibility is that the observed increase in MAGL with omega-3 intake may be associated with a rise in arachidonic acid (AA). AA is known to sustain LTP at perforant path synapses [58], and its combination with mGluR1 activation induces a rapid, long-lasting potentiation [59]. Conversely, LTP induction at perforant path synapses prevents mGluR and AA synergism from triggering presynaptic changes that enhance glutamate release, further underscoring their roles in LTP at these synapses [60].

The eCB-dependent LTP was unaffected by the NMDA receptor antagonist D-AP5, indicating that NMDA receptors were not involved in synaptic potentiation following LFS in omega-3-fed mice, even though LTP at MPP synapses typically requires NMDA receptor activation [61,62]. Classical studies suggest that short-term potentiation (STP) is expressed at presynaptic sites and involves retrograde messengers [63]. Interestingly, STP and LTP were shown to be mediated by different NMDAR subunits, with STP being more resistant to D-AP5 than LTP [64,65]. Thus, the sustained fEPSP potentiation observed over a 30-min period after MPP LFS in omega-3-fed mice might be related to STP.

However, in our previous study, we found that D-AP5 blocked potentiation following LFS in CB1 knockout mice, which suggests an underlying increase in glutamate release and subsequent NMDA receptor activation [26,66]. Additionally, the blockade of MPP-LTP by CPCCoEt and the partial blockade by MPEP indicate that MPP-LTP relies on mGluR1 and, to a lesser extent, mGluR5. The distinct recovery kinetics after LFS in the presence of CPCCoEt or MPEP suggest that both group I mGluRs play unique roles in eCB-LTP at MPP-granule cell synapses in omega-3-fed mice. Specifically, CPCCoEt affects both the induction and late phases, while MPEP influences only the late phase of fEPSP potentiation. Although both mGluR1 and mGluR5 are required for LTD elicited by the same LFS at MPP synapses, our previous observations indicated a predominance of mGluR5 over mGluR1, as only mGluR5 was necessary for the initial eCB-eLTD phase [26].

In omega-3-fed mice, MPP-LTP was independent of TRPV1 and AEA. Interestingly, in mice exposed to environmental enrichment, a switch from MPP-LTD to LTP was previously observed, which required TRPV1 activation and AEA but was independent of CB1 receptors, group I mGluRs, and 2-AG [39]. This suggests that the recruitment of molecular components underlying the synaptic plasticity switch at MPP synapses varies depending on the type of external factor—nutritional or environmental.

The omega-3 diet also resulted in a substantial (~40%) decrease in PSD-95 levels. This postsynaptic protein is crucial for structural synaptic plasticity and the clustering of glutamate receptor subunits at excitatory synapses [67,68,69]. Indeed, PSD-95 knockin and knockout mice exhibit altered NMDAR and AMPAR subunit expression and function [69,70]. Interestingly, LTP was enhanced at MPP synapses in adult PSD-95 knockin mice, possibly due to compensatory mechanisms [70]. Similar compensatory processes may also contribute to the MPP-LTP observed in omega-3-fed mice. Notably, the significant decrease in hippocampal PSD-95 resulting from the omega-3-enriched diet did not negatively affect recognition memory, consistent with previous findings in PSD-95-deficient mice [69,70].

Mice on an omega-3-enriched diet exhibited reduced exploration and significantly improved discrimination in the NORT. These findings align with reports that EPA and DHA supplementation enhances various types of memory, including recognition memory [71,72,73,74]. Additionally, increased eCB signaling has been shown to restore emotional and cognitive functions and rescue eCB-dependent synaptic plasticity impaired by omega-3 PUFA deficiency in brain regions related to mood and cognition [14,75,76,77]. Omega-3 PUFAs, particularly EPA and its derivatives, influence cAMP levels by modulating the activity of cAMP-hydrolyzing phosphodiesterase (PDE) [78,79,80,81]. Since PDE-4 inhibition has been shown to restore recognition memory [82], it is possible that cAMP modulation through PDE activity could be one mechanism by which omega-3 PUFAs enhance recognition memory.

We also found that omega-3 intake during young adulthood produced an anxiolytic-like effect, consistent with previous findings of increased anxiety-like behaviors associated with omega-3 deficiency [76] and, conversely, with the anxiolytic effects of EPA and DHA supplementation in both pathological and healthy conditions [73,83,84,85]. The mood effects observed in our model of omega-3 intake may be linked to alterations in membrane phospholipids and subsequent changes in eCB levels, particularly 2-AG [30].

## 5. Conclusions

An omega-3-enriched diet alters the synaptosomal expression of CB1 receptors, 2-AG synthesizing and degrading enzymes, as well as other pre- and postsynaptic proteins in the hippocampus, supporting MPP synaptic potentiation along with cognitive and mood enhancements. Our biochemical, physiological, cognitive, and behavioral findings further underscore the positive impact of omega-3 PUFAs on brain health.

## Figures and Tables

**Table 1:** Comparison of fatty acid (FA) composition in standard vs. omega-3 (*n*-3)-enriched diet.

	Standard Diet		*n*-3-Enriched Diet	
Fats (%)	4.0		5.9	
	mg/kg	% of Total Fats	mg/kg	% of Total Fats
SFA	6000	15.0	11,867	20.1
PUFA	21,000	52.5	31,129	52.8
*n*-3	1000	2.5	5437	9.2
*n*-6	20,000	50.0	25,679	43.5
Ratio *n*-6/*n*-3	-	20.0	-	4.7
EPA	0	0	1325	2.2
DHA	0	0	899	1.5

**Table 2:** Primary antibodies used in Western blot assays.

Antibody	Dilution	Species and Clonality	Isotype	Immunizing Antigen	Source, Cat.
CB1	1:1000	Goat polyclonal	Serum	Mouse CB1, *C*-terminal 31 aa (NM007726)	Nittobo Medical Co. (Tokyo, Japan), CB1-Go-Af450
Crip1a	1:500	Rabbit polyclonal	IgG	Peptide mapping within an internal region of CRIP1 of human origin	Santa Cruz Biotechnology, Inc. (Dallas, TX, USA), sc-137401
Gαo	1:200	Mouse monoclonal	IgG_1_ kappa light chain	Raised against Gαo of bovine origin	Santa Cruz Biotechnology, Inc. (Dallas, TX, USA), sc-13532
Gαi1	1:200	Mouse monoclonal	IgG_2b_ kappa light chain	Raised against Gαi1 of rat origin	Santa Cruz Biotechnology, Inc. (Dallas, TX, USA), sc-13533
Gαi2	1:200	Mouse monoclonal	IgG_2b_ kappa light chain	Raised against Gαi2 of rat origin	Santa Cruz Biotechnology, Inc. (Dallas, TX, USA), sc-13534
Gαi3	1:200	Mouse monoclonal	IgG_3_ kappa light chain	Epitope mapping between amino acids 339–354 at the *C*-terminus of Gαi3 of rat origin	Santa Cruz Biotechnology, Inc. (Dallas, TX, USA), sc-365422
PLCβ1	1:2000	Sheep Polyclonal	IgG	*E. coli*-derived recombinant human PLC-β1 Lys27-Met245	Novus Biologicals (Centennial, CO, USA), AF4466
DAGLα	1:1000	Rabbit polyclonal	Serum	Mouse DGL-α, *C*-terminal 42 aa (NM198114)	Nittobo Medical Co. (Tokyo, Japan), DGLa-Rb-Af380
DAGLβ	1:2000	Rabbit monoclonal	IgG	Synthetic peptide corresponding to residues surrounding Leu505 of human DGL-β protein	Cell Signaling Technology, Inc. (Danvers, MA, USA), 12574
MAGL	1:1000	Rabbit polyclonal	Serum	Mouse MGL, 1–35aa (NM_011844)	Nittobo Medical Co. (Tokyo, Japan), MGL-Rb-Af200
PSD-95	1:2500	Goat polyclonal	IgG	Synthetic peptide derived from residues 1–100 of mouse PSD95	Abcam (Cambridge, UK), ab12093
SNAP-25	1:10,000	Rabbit polyclonal	IgG	Synthetic peptide within residues 150 to the *C*-terminus of human SNAP25	Abcam (Cambridge, UK), ab41455

**Table 3:** Drugs used in electrophysiological experiments.

Drug	Action	Dilution (µM)
AM251	CB1 antagonist	4
AMG9810	TRPV1 antagonist	3
MPEP	mGluR5 antagonist	10
THL	DGL inhibitor	10
RHC 80267	DGL inhibitor	100
D-AP5	NMDA antagonist	50
Latrunculin A	Actin assembly inhibitor	500
ω-conotoxin GVIA	*N*-type Ca^2+^ channels blocker	1
CPCCoEt	mGluR1 antagonist	50
LEI401	NAPE-PLD inhibitor	10
WIN-2	CB1 agonist	5

**Table 4:** Relative protein expression determined by Western blot analysis. Values represent the ratio of slopes between control and omega-3 (*n*-3) groups presented as mean ± SEM of independent experiments (the number of experiments performed for each protein is denoted), using whole homogenate (wH) or synaptosomal fractions obtained from two fractionation procedures and including hippocampal pools from at least six adult mice per fractionation procedure. Regression analysis of immunoreactive signal values (integrated optical density) corresponding to increasing amounts of synaptosomal total protein provided the equation of a simple linear regression model. The resulting slopes of the equations were subsequently used to determine the relative protein expression defined as the ratio of the slopes (*n*-3/control). Statistical analysis: paired ratio *t*-test; * *p* < 0.05, ** *p* < 0.01, *** *p* < 0.001.

Fraction	Protein	*n*-3
wH	CB1	0.972 ± 0.046 (n = 3)
	Crip1a	1.024 ± 0.016 (n = 3)
Synaptosomes	CB1	1.333 ± 0.065 ** (n = 8)
	Crip1a	1.487 ± 0.095 *** (n = 11)
	Gαo	0.771 ± 0.057 * (n = 4)
	Gαi1	0.824 ± 0.081 (n = 5)
	Gαi2	0.924 ± 0.059 (n = 4)
	Gαi3	0.779 ± 0.075 * (n = 7)
	PLCβ1	1.263 ± 0.059 ** (n = 8)
	DAGLα	0.635 ± 0.059 ** (n = 7)
	DAGLβ	1.205 ± 0.064 (n = 7)
	MAGL	1.250 ± 0.055 *** (n = 7)
	SNAP-25	0.734 ± 0.076 * (n = 5)
	PSD-95	0.650 ± 0.082 * (n = 5)

**Table 5:** Concentration–response curve for CP 55,940-stimulated specific [^35^S]GTPγS binding in hippocampal synaptosomes from control and omega-3 (*n*-3) mice. Values correspond to the means ± SEM of three independent experiments, using synaptosome membranes obtained from two fractionation procedures and including hippocampal pools from at least six adult mice per fractionation procedure. Statistical analysis: paired *t*-test.

	Control	*n*-3
E_max_	156.90 ± 12.80	159.00 ± 8.56
pEC_50_	7.03 ± 0.07	7.13 ± 0.08

## Data Availability

The raw data supporting the conclusions of this article will be made available by the authors on request.
